# Post-thyroidectomy anterior neck dysfunction: clinical phenotypes, mechanisms, and comprehensive management

**DOI:** 10.3389/fendo.2026.1859968

**Published:** 2026-08-20

**Authors:** Xiaokang Liu, Yu Li, Qiang Ma, Lijuan An, Liang Guo

**Affiliations:** Department of General Surgery, Lanzhou University Second Hospital, Lanzhou University, Lanzhou, China

**Keywords:** adhesion, anterior neck dysfunction, rehabilitation, swallowing dysfunction, thyroidectomy, voice disorder

## Abstract

Thyroid surgery is generally safe; however, satisfactory postoperative recovery is not determined solely by traditional complications such as recurrent laryngeal nerve injury, bleeding, or hypocalcemia. A subset of patients develop interrelated symptoms—including voice changes, swallowing discomfort, anterior neck pain or tightness, restricted cervical mobility, and sensory abnormalities—that are considered here within a proposed framework of post-thyroidectomy anterior neck dysfunction. In this review, the term is used as a working conceptual framework rather than a formally established diagnostic entity. Plausible mechanisms include neural injury, anterior neck muscle injury, restricted laryngotracheal motion, adhesion and fibrosis, and altered sensory input. Using a structured narrative synthesis of the available literature, we summarize the clinical phenotypes, natural history, potential mechanisms, risk factors, assessment tools, and prevention and treatment strategies relevant to this framework.

## 1 Introduction

Advances in surgical techniques, neural monitoring, energy devices, and endoscopic approaches have markedly reduced the rate of major complications after thyroid surgery. However, postoperative recovery is not determined solely by the presence or absence of conventionally defined complications. Existing studies show that some patients develop voice changes, swallowing discomfort, anterior neck tightness, restricted mobility, and local numbness or paresthesia even without definite recurrent laryngeal nerve injury. Although these problems are rarely life-threatening, they may exert a sustained adverse effect on communication, work performance, and quality of life (4, 6, 9, 12, 16, 33). Functional burden may also be compounded by traditional complications such as permanent hypoparathyroidism, which has been associated with poorer self-reported quality of life and voice outcomes (52).

For many years, functional problems after thyroidectomy have been investigated in a fragmented manner across disciplines. Voice abnormalities have often been reduced to neural complications; swallowing discomfort has frequently been regarded as a transient postoperative response; and neck pain, traction, and scar-related symptoms have usually been classified as part of routine recovery. This fragmented approach does not fully explain persistent functional impairment in patients without clear neural injury, nor does it support integrated management centered on patient-reported symptom burden (5, 7, 35). This review therefore uses impairment of the anterior neck functional unit as an organizing framework for these manifestations. Throughout the manuscript, post-thyroidectomy anterior neck dysfunction denotes a proposed conceptual synthesis intended to connect related postoperative phenotypes; it should not be interpreted as a formally established diagnosis or universally accepted syndrome. The review develops this framework for clinical interpretation and future study (28, 36, 66).

### 1.1 Literature search and selection

This narrative review was developed from a structured search and thematic synthesis of the PubMed literature. PubMed was the bibliographic database used. Searches performed during manuscript preparation combined “thyroidectomy” or “thyroid surgery” with terms relating to voice or dysphonia, swallowing or dysphagia, anterior neck pain/discomfort/tightness, cervical mobility, sensation, scar or adhesion, rehabilitation, and quality of life. The literature set was updated in July 2026. Because the review was developed iteratively, no prospectively registered protocol or single prespecified Boolean search string was used.

An initial corpus of 455 records was screened by title and abstract, and 104 were classified as directly relevant to the review question. The final working set comprised 70 publications, with one additional 2026 systematic review added during revision. Eligible sources included original clinical studies, randomized trials, systematic or scoping reviews, meta-analyses, clinical guidelines, and validation studies that directly addressed post-thyroidectomy functional outcomes or informed the proposed mechanistic and assessment framework. Studies were excluded if they were unrelated to thyroid surgery, focused exclusively on traditional complications without functional outcomes, or did not materially inform the review questions. During corpus refinement, direct relevance and recency were prioritized, and a five-year journal impact factor greater than 2 was used as a pragmatic journal-level filter. This filter was not used to grade individual studies and is acknowledged as a potential source of selection bias. No formal risk-of-bias tool or quantitative pooling was applied.

## 2 Conceptual definition of the proposed framework

From an anatomical and functional perspective, the anterior neck is not a single structure. Rather, it is a functional network composed of the recurrent and superior laryngeal nerves, the infrahyoid musculature, the laryngotracheal complex, the anterior cervical fascia and soft-tissue glide planes, the superficial cutaneous nerves, and the scar system. Disturbance of any component of this network during thyroidectomy may manifest as voice changes, swallowing dysfunction, local pain or tightness, restricted movement, sensory abnormalities, and reduced quality of life. Accordingly, we propose post-thyroidectomy anterior neck dysfunction as a working conceptual framework for functional disturbances that may result from disruption of the neural, muscular, fascial, soft-tissue glide, and superficial sensory systems after thyroidectomy (1, 4, 6, 8, 9, 49). It is not presented as a formally validated diagnostic entity or consensus syndrome.

The proposed framework encompasses six broad phenotype domains: voice dysfunction, swallowing dysfunction, anterior neck pain or tightness, restricted cervical mobility, sensory abnormalities, and scar- and adhesion-related discomfort (19, 22, 37, 42, 53, 56). These phenotypes are interrelated rather than independent. Voice changes are often accompanied by throat discomfort or traction in the neck. Swallowing dysfunction may be associated with restricted laryngotracheal elevation and local adhesion. Sensory abnormalities may further amplify symptom perception in susceptible individuals (33, 50, 60).

**Figure 1:**
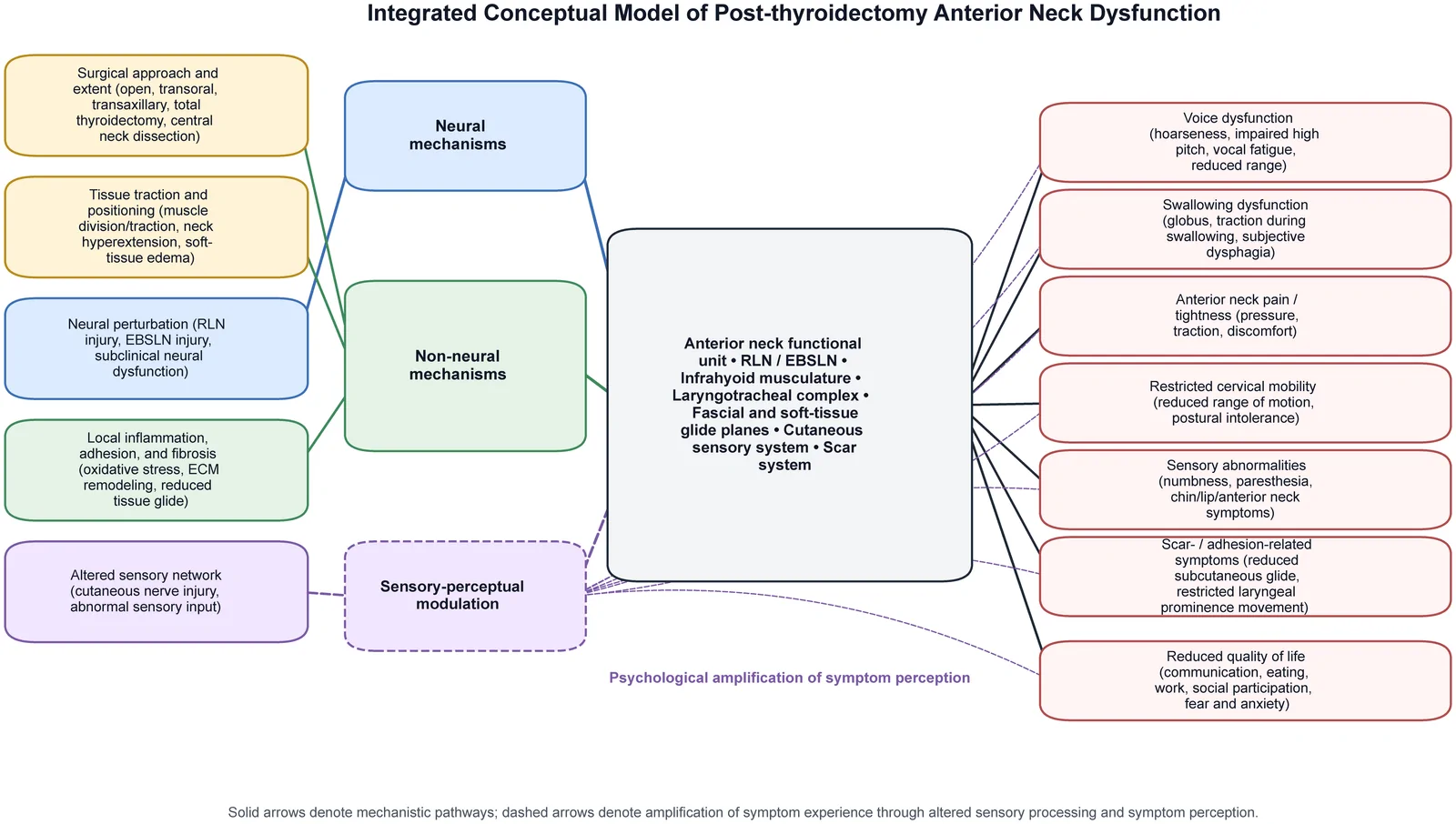
Proposed integrated conceptual model of post-thyroidectomy anterior neck dysfunction. This figure presents post-thyroidectomy anterior neck dysfunction as a proposed working framework rather than a formally established diagnostic entity. The left panel groups principal perioperative drivers, including surgical approach and extent, tissue traction and positioning, neural perturbation, local inflammation/adhesion/fibrosis, and altered sensory input. These drivers may converge through neural and non-neural pathways, with sensory-perceptual modulation acting as a possible overlay that amplifies symptom burden. The central functional unit includes the recurrent and superior laryngeal nerves, infrahyoid musculature, the laryngotracheal complex, fascial and soft-tissue glide planes, the cutaneous sensory system, and the scar system. The right panel summarizes the major clinical outputs. Arrows indicate proposed relationships and should not be interpreted as proof of causality.

**Figure 1** summarizes the proposed integrated framework.

## 3 Clinical phenotypes and natural history

### 3.1 Voice dysfunction

Most available studies have focused on postoperative voice dysfunction. Even in patients without injury to the recurrent laryngeal nerve or the external branch of the superior laryngeal nerve, clinically relevant subjective voice complaints may occur after surgery. Common manifestations include hoarseness, impaired high-pitched phonation, vocal fatigue, voice instability, and reduced pitch range (2, 4, 11, 12, 16, 37).

Voice dysfunction is usually most prominent in the early postoperative period. Most patients improve over several weeks to months, but a subset continue to report residual symptoms at 6 months, 1 year, or even longer. Long-term follow-up suggests that older age, total thyroidectomy, and larger preoperative thyroid volume may delay recovery. By contrast, a 10-year follow-up study did not demonstrate permanent or progressive deterioration in voice function, indicating that persistent symptoms are not necessarily equivalent to irreversible structural injury (16, 34, 40).

### 3.2 Swallowing dysfunction

Postoperative swallowing discomfort is also common, particularly in the early period after surgery. Typical complaints include globus sensation, traction during swallowing, impaired bolus passage, and subjective dysphagia. Most patients gradually return toward their preoperative state within 2–3 months, although some continue to experience swallowing discomfort at 6 months or even 1 year after surgery (3, 4, 6, 50, 71). A recent systematic review similarly described an early postoperative peak, recovery toward baseline by approximately 2–3 months in most patients, and persistent symptoms in a minority, while emphasizing substantial heterogeneity in definitions, assessment methods, and follow-up (71).

A key feature of postoperative swallowing dysfunction is the discrepancy between subjective symptoms and objective findings. Some patients report marked dysphagia, whereas videofluoroscopic swallowing study (VFSS) and flexible endoscopic evaluation of swallowing (FEES) may not demonstrate aspiration or obvious transport impairment (50, 54). These observations suggest that post-thyroidectomy swallowing dysfunction may not solely reflect impaired bolus transit. Potential contributors include restricted motion of the laryngotracheal complex, anterior neck adhesion, abnormal sensory input, and pain-related hypervigilance; however, the relative contribution of each mechanism remains uncertain (3, 8, 71).

### 3.3 Anterior neck pain, tightness, and restricted mobility

Anterior neck pain, pressure, traction, and reduced range of motion most directly reflect the concept of anterior neck dysfunction. Patients commonly describe a tight neck, difficulty extending the head, traction beneath the incision during swallowing, or soreness in the neck and shoulder region. These complaints are difficult to explain within the traditional framework of postoperative complications (13, 19, 22, 36, 67).

These symptoms are usually most pronounced in the early postoperative period and tend to improve as soft-tissue edema resolves and the scar matures, although they may persist for weeks to months in some patients. In clinical practice, postoperative recovery should therefore not be judged solely by the presence or absence of pain; range of motion, traction, and functional tolerance also merit attention (26, 36, 67).

### 3.4 Sensory abnormalities and scar-/adhesion-related symptoms

With the increasing adoption of endoscopic and robotic thyroidectomy, postoperative sensory abnormalities have become more clinically relevant. After transoral approaches, altered sensation of the chin, lower lip, and upper neck is relatively common. Most cases improve within a few months, but subjective recovery does not always parallel normalization of objectively measured sensory thresholds (9, 60). Thus, postoperative sensory dysfunction may reflect not only peripheral nerve conduction abnormalities but also local sensory nerve remodeling and central symptom perception (9, 56, 60).

Scar- and adhesion-related symptoms also occur after open surgery. Patients may report traction of the incision during swallowing, impaired mobility of the laryngeal prominence, reduced subcutaneous glide, and persistent local discomfort. Clinical studies of anti-adhesion materials support a plausible link between postoperative adhesion, impaired tissue glide, and local symptoms, but material types, outcome definitions, and follow-up vary substantially (8, 38, 44, 49). Experimental work further supports inflammation and fibrosis as biologically plausible contributors (65). Anti-adhesion materials may improve selected early outcomes, but durable clinical benefit remains uncertain.

**Figure 2:**
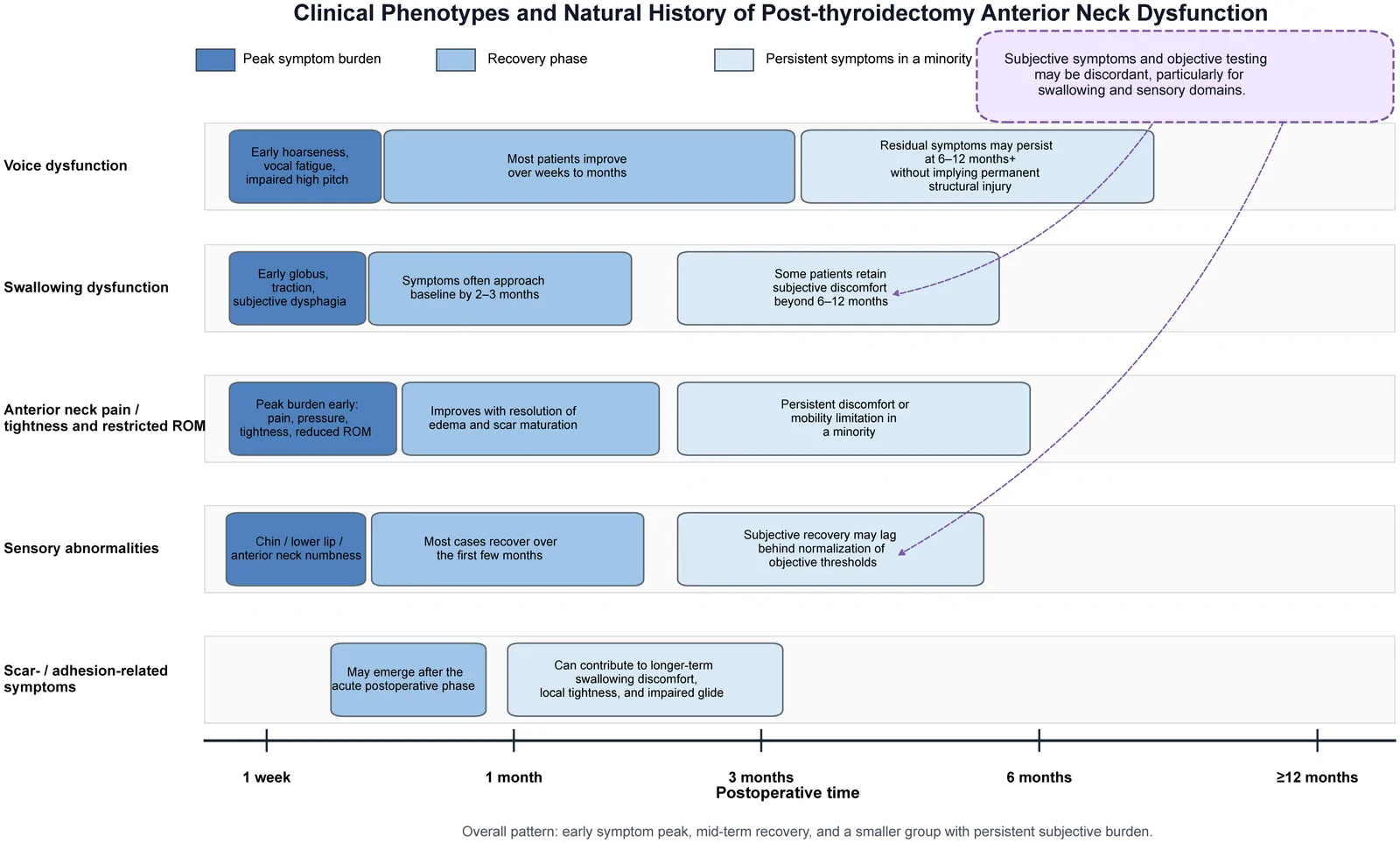
Illustrative natural history of post-thyroidectomy anterior neck dysfunction. This timeline provides an illustrative synthesis of symptom evolution after thyroidectomy and is not based on pooled time-to-event estimates. Voice dysfunction, swallowing dysfunction, anterior neck pain/tightness with reduced range of motion, and sensory abnormalities often peak in the early postoperative period and then improve over weeks to months. Scar- and adhesion-related symptoms may become more apparent after the acute postoperative phase and may contribute to longer-term local discomfort and impaired tissue glide. Subjective symptoms and objective test findings may be discordant, especially in the swallowing and sensory domains. The time course should therefore be interpreted as a general pattern derived from heterogeneous studies rather than a uniform trajectory for all patients.

**Figure 2** illustrates the broad clinical spectrum and natural-history pattern derived from the reviewed literature.

## 4 Effects of surgical approach and extent on anterior neck function

Available evidence suggests that surgical approach may influence postoperative function in addition to cosmetic outcomes. Supraclavicular, lateral cervical, transoral, transaxillary, and intermuscular approaches may reduce disruption of selected midline tissues or offer advantages in postoperative neck discomfort, swallowing experience, or voice-related questionnaire scores (20, 23, 31, 42, 45). These findings indicate that the incision site and anatomical access route may contribute to the distribution of functional burden, although direct comparisons remain heterogeneous.

Current evidence does not support designating any single approach as universally optimal. Some studies report more favorable early swallowing-related quality of life after transoral approaches (14, 20, 42), whereas other comparisons show broadly similar overall recovery between robotic and conventional surgery. Remote-access approaches may also introduce route-specific sensory morbidity, including chin, lower-lip, or anterior chest sensory change (9, 56, 60). The clinically relevant question is therefore not whether one approach is intrinsically “better” or “worse,” but how the access route alters the distribution and time course of functional burden.

Compared with the surgical approach, the extent of surgery appears to correlate more strongly with functional abnormalities. Total thyroidectomy, central neck dissection, larger thyroid volume or weight, and longer operative time have been associated with greater postoperative voice or swallowing dysfunction (2, 12, 37, 50, 51). These associations may reflect broader tissue manipulation, neural stress, edema, and disruption of soft-tissue glide planes.

## 5 Potential mechanisms: from neural injury to dysfunction of the anterior neck functional unit

### 5.1 Neural mechanisms

Recurrent laryngeal nerve injury can cause unilateral vocal fold paralysis, glottic insufficiency, and overt voice dysfunction, and in some patients may secondarily impair swallowing (24, 46). Injury to the external branch of the superior laryngeal nerve weakens cricothyroid muscle function and may manifest as impaired high-pitched phonation, reduced pitch range, impaired pitch control, and vocal fatigue, even when laryngoscopic findings are not striking (29, 39).

However, neural mechanisms do not appear to explain all postoperative symptoms. A substantial proportion of patients without definite recurrent laryngeal nerve or external branch of the superior laryngeal nerve injury still report marked voice, swallowing, or anterior neck complaints (4, 6, 12, 16). Within the proposed framework, neural injury is therefore considered one potential component rather than a complete explanation.

### 5.2 Non-neural mechanisms

Division and traction of the anterior strap muscles may alter myofascial function and contribute to non-neural symptoms. Although isolated division of the sternothyroid muscle does not necessarily lead directly to voice or swallowing dysfunction, more extensive muscle traction, disruption of the cervical midline connective tissue, and local edema may affect the mobility and tension balance of the laryngotracheal complex (23, 30, 48).

Positioning is another frequently overlooked factor. Intraoperative neck hyperextension has been associated with greater neck pain within 24 hours after surgery. It may also contribute to postoperative tightness and restricted mobility through alterations in anterior neck soft-tissue tension and posture-related myofascial stress (22). Perioperative functional-protection strategies may therefore include careful positioning in addition to exposure, hemostasis, and neural preservation.

Adhesion formation and fibrosis are plausible non-neural mechanisms. Local surgical trauma may trigger inflammation, oxidative stress, extracellular matrix remodeling, and fibrosis, thereby reducing soft-tissue glide around the laryngotracheal complex and causing abnormal traction during swallowing. These processes may contribute to voice symptoms, swallowing complaints, and local discomfort, although the clinical contribution of each pathway has not been quantified (8, 49, 65).

Reflux-like laryngopharyngeal symptoms and reflux finding scores may worsen transiently after thyroidectomy, but current evidence does not establish laryngopharyngeal reflux as a unifying cause of postoperative voice or swallowing symptoms (7, 21).

### 5.3 Abnormal sensory input and amplification of symptom perception

The sensory system may also contribute to postoperative symptom generation. After transoral or transthoracic endoscopic thyroidectomy, numbness of the chin, lower lip, anterior chest, or anterior neck skin suggests altered somatosensory input associated with cutaneous nerve injury. Such sensory disturbances may affect speaking, swallowing, and neck movement (9, 56, 60).

Quality-of-life studies further indicate that voice changes and persistent neck discomfort may be associated with restricted social participation, anxiety about symptoms, and fear of disease recurrence, which may prolong subjective recovery (33). If patients have limited understanding of possible postoperative voice and functional abnormalities, even mild-to-moderate symptoms may be perceived as more severe (5, 35). Accordingly, altered sensory input and symptom-perception amplification are included as plausible modulators within the proposed framework rather than established causal pathways.

## 6 Risk factors and high-risk populations

Older age has been associated with pre-existing and postoperative swallowing burden in older adults (27) and with slower recovery of non-neural voice changes (34). Higher body mass index has been associated with poorer postoperative voice measures in some cohorts (12). These findings arise from heterogeneous observational studies and should not be interpreted as validated causal predictors.

In addition to patient-related characteristics, surgery-related factors have been associated with postoperative functional outcomes. Total thyroidectomy, central neck dissection, larger gland volume or weight, longer operative time, and more extensive tissue dissection have each been linked to an increased risk of postoperative voice or swallowing dysfunction (12, 37, 50, 51). Attenuation of the intraoperative signal from the external branch of the superior laryngeal nerve may also serve as a warning sign, suggesting that meticulous neural preservation should not be limited to the recurrent laryngeal nerve (39).

Furthermore, in patients with high occupational voice demands or strong dependence on verbal communication, even mild impairment of high-pitched phonation, vocal fatigue, or pharyngeal discomfort may translate into clinically meaningful disability. Individualized risk assessment should therefore consider not only the disease and the procedure, but also the patient’s occupational and lifestyle requirements (5, 35).

## 7 Assessment framework

Because postoperative functional problems first manifest as subjective symptoms, patient-reported outcome measures occupy a foundational role in assessment. The Voice Handicap Index (VHI) and Voice Handicap Index-10 (VHI-10) are the most widely used subjective instruments. The Thyroidectomy-related Voice and Symptom Questionnaire (TVSQ) and Thyroidectomy-related Voice Questionnaire (TVQ) integrate voice changes with laryngopharyngeal and anterior neck discomfort, making them particularly relevant to this review (1, 10, 17, 18, 69). The Swallowing Quality of Life questionnaire (SWAL-QOL) assesses the impact of dysphagia on daily life (20, 42), whereas THYCA-QoL captures broader physical, psychological, and social effects in patients with thyroid cancer (53). The Functional Oral Intake Scale may complement instrumental assessment of oral intake, although the cited validation was not specific to thyroidectomy (63).

Objective testing is also essential. Common voice measures include acoustic parameters, maximum phonation time, frequency range, cepstral peak prominence, and laryngoscopic assessment of vocal fold vibration (15, 32, 46). Machine-learning models have also been explored for prediction or characterization of postoperative voice recovery (25, 47). For swallowing, FEES and VFSS are the most commonly used instrumental assessments (3, 50, 54, 71). Selected studies have also measured cervical range of motion, visualized cervical adhesion, or quantified cutaneous sensory thresholds (9, 19, 49, 60). A recent systematic review confirmed wide variation in dysphagia definitions, patient-reported instruments, objective tests, and follow-up schedules (71). This heterogeneity limits cross-study comparison and precludes a single universally accepted assessment protocol (3, 7, 26, 28, 71).

## 8 Prevention and treatment strategies

**Figure 3:**
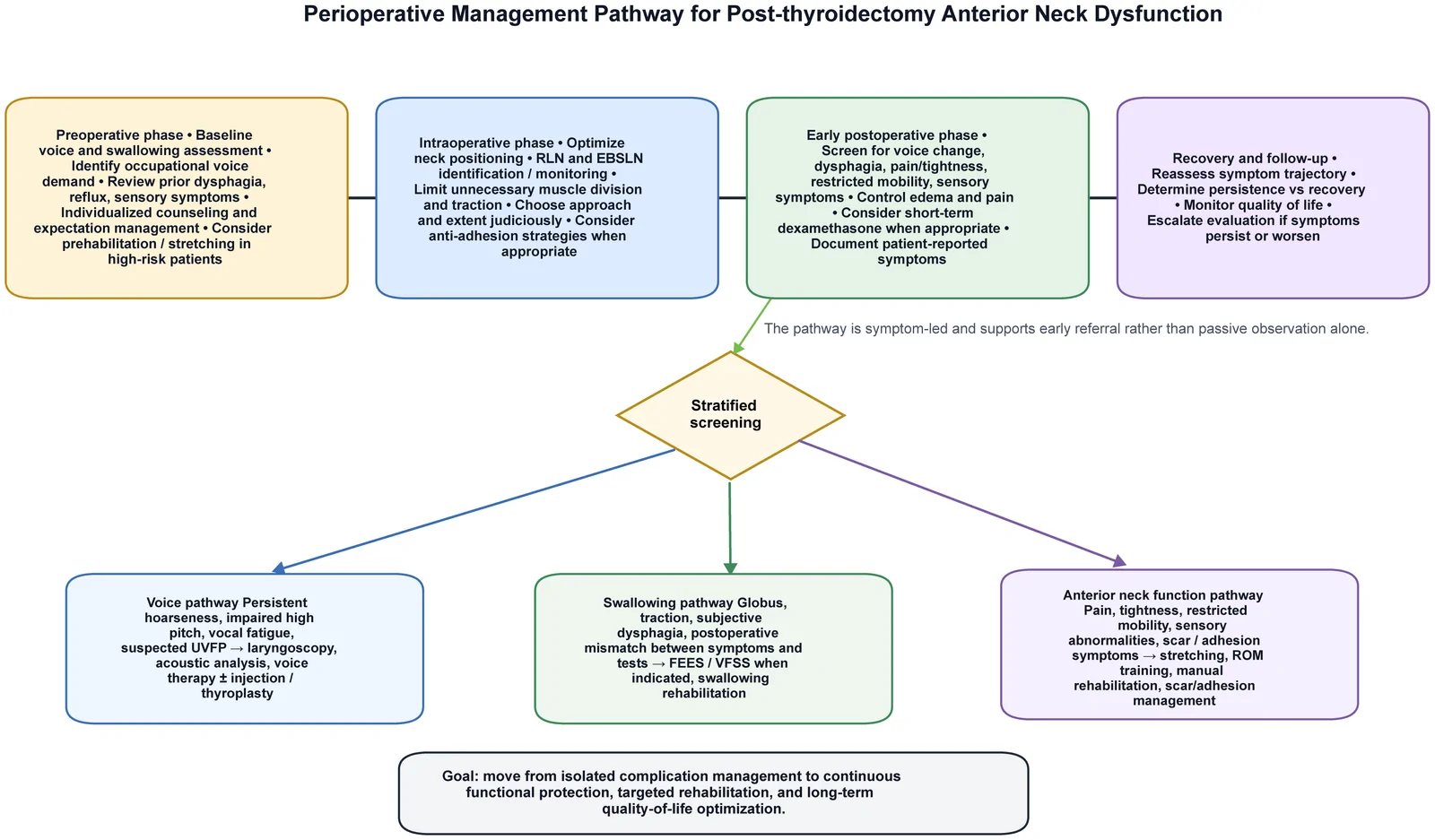
Proposed perioperative management pathway for post-thyroidectomy anterior neck dysfunction. This figure translates the review into a proposed perioperative management pathway. The process begins with preoperative baseline assessment, individualized counseling, and selective prehabilitation, followed by intraoperative considerations including neural identification and monitoring, minimization of unnecessary muscle division and traction, optimization of neck positioning, protection of relevant sensory nerves, and selective consideration of anti-adhesion measures. In the early postoperative phase, symptom-led screening serves as the entry point for triage and targeted referral. The pathway is intended as a clinically testable framework rather than a formal guideline; the strength of evidence varies across individual interventions.

A perioperative management framework for post-thyroidectomy anterior neck dysfunction is shown in **Figure 3**.

### 8.1 Preoperative strategies

Preoperative management is the starting point for functional protection. Baseline voice and swallowing assessment may be performed when clinically feasible, with documentation of VHI or TVSQ as needed to facilitate postoperative comparison. Clinicians should also clarify the patient’s occupational voice demands, prior swallowing status, and main concerns about postoperative recovery to provide individualized risk communication (5, 18, 28, 35). For higher-risk patients, a clearly defined preoperative baseline may support expectation management and timely postoperative referral for rehabilitation.

Prehabilitation has recently attracted increasing attention. A recent randomized trial suggested that starting neck stretching before surgery may reduce early neck pain compared with postoperative exercise alone, although effects on VHI and overall quality of life were limited (67). Subsequent correspondence and the broader evidence base underscore that the optimal timing and content remain unsettled (36, 70). Because the intervention literature is small and heterogeneous, prehabilitation should be considered selectively rather than as a universal standard.

### 8.2 Intraoperative strategies

The core intraoperative goal is to preserve the anterior neck functional unit to the greatest extent compatible with safe and oncologically appropriate surgery. In addition to routine preservation of the recurrent laryngeal nerve, identification and monitoring of the external branch of the superior laryngeal nerve may be particularly relevant when disease involves the superior pole, upper-pole dissection is technically demanding, or a wider resection is required (29, 39). In selected patients with thyroid cancer who require recurrent laryngeal nerve resection to achieve an R0 resection, immediate nerve reconstruction may create more favorable conditions for postoperative voice recovery (59).

Current evidence does not identify a single technique that prevents postoperative anterior neck dysfunction. Nevertheless, surgeons can apply several function-preserving principles: select an approach according to oncologic, technical, and patient-specific considerations; avoid unnecessary neck hyperextension; identify and protect the recurrent laryngeal nerve and external branch of the superior laryngeal nerve; minimize unnecessary strap-muscle division, traction, and dissection; preserve midline connective tissues and soft-tissue glide planes when feasible; and protect the mental or supraclavicular nerves during relevant remote-access procedures (9, 22, 23, 30, 31, 39, 45, 48, 49, 56, 60). These are practical considerations rather than a validated intervention bundle.

The selective use of anti-adhesion materials is another potentially beneficial intraoperative strategy. Available studies suggest that several agents are generally safe and may reduce adhesion through anti-inflammatory, antioxidant, or antifibrotic effects, with possible early benefits in swallowing symptoms, voice-related symptoms, or local tissue glide (8, 38, 44, 49, 65). However, studies differ substantially in material composition, comparator, endpoint, and follow-up, and long-term clinically meaningful benefit has not been established. Routine use solely to prevent postoperative functional symptoms cannot yet be recommended.

### 8.3 Early postoperative management

The main goals of early postoperative management are to reduce edema, control pain, and promote functional recovery. Dexamethasone may improve subjective voice dysfunction within the first 48 hours after surgery, although durable objective benefit and efficacy in patients with definite neural injury remain uncertain (43, 55). Local incisional infiltration, cervical plexus block, and multimodal analgesia may reduce early pain or discomfort, but the available protocols and outcome measures are heterogeneous (13).

One randomized trial evaluated alpha-lipoic acid plus B vitamins for postoperative dysphonia. The overall findings did not establish a consistent benefit, and routine use cannot be recommended (62).

### 8.4 Rehabilitation

Rehabilitation is a promising component of management within the proposed framework. Available studies suggest that neck stretching, mobility training, oropharyngeal swallowing exercises, and comprehensive rehabilitation programs may improve neck pain, range of motion, or swallowing-related quality of life (19, 26, 36, 66). However, many studies are small or single-center, and intervention timing, frequency, content, comparators, and follow-up vary substantially. Systematic reviews and trial-level reports therefore support a favorable but still uncertain signal rather than a standardized protocol (26, 36, 41, 64, 67, 71).

For selected patients with unilateral vocal fold paralysis, voice therapy, hyaluronic acid injection, fat injection, and thyroplasty may improve voice outcomes. In patients unable to attend in-person therapy, telerehabilitation has shown feasibility and potentially useful outcomes (24, 57). Rare severe dysphagia may require individualized multidisciplinary support, as illustrated by case-based experience (68). Management should be guided by documented impairment, symptom trajectory, and specialist assessment rather than passive observation alone; a clear process for early screening and targeted referral is therefore appropriate.

## 9 Discussion

Research on postoperative functional outcomes after thyroidectomy is shifting from a narrow focus on neural complications toward integrated assessment centered on patient-reported symptoms and multidimensional outcomes. Voice and swallowing are the two domains with the most concentrated evidence; however, discussing them separately by organ system does not fully account for persistent pain, tightness, traction, sensory abnormalities, and reduced quality of life in patients without definite neural injury (3, 4, 6, 9, 16). The anterior neck functional unit is therefore proposed here as a working framework that may help organize these phenotypes and integrate previously fragmented evidence related to neural injury, myofascial disturbance, adhesion, and sensory processing.

Non-neural mechanisms deserve greater consideration alongside neural protection, although the causal evidence is less mature. Studies of anti-adhesion materials, positioning, intraoperative muscle preservation, laryngotracheal glide, and perioperative rehabilitation collectively suggest that thyroidectomy may alter the mechanical and sensory environment of the anterior neck (8, 22, 49, 65, 66). These observations support the proposed framework, but they do not establish that any single non-neural pathway is necessary or sufficient for persistent postoperative symptoms.

The discrepancy between subjective symptoms and objective findings is another phenomenon that requires special attention (50, 54, 60). Future studies should not rely exclusively on laryngoscopy, FEES, or acoustic parameters, but should integrate sensory threshold testing, cervical range-of-motion assessment, soft-tissue glide evaluation, and long-term quality-of-life measures. Only by acknowledging and addressing this discrepancy methodologically can we explain why some patients have only mild objective abnormalities yet substantial symptom burden.

From a translational perspective, the key goal is not to prove the absolute superiority of a single surgical approach or intervention, but to develop a continuous and testable management pathway spanning the preoperative, intraoperative, and postoperative periods. Preoperative baseline assessment and counseling may identify higher-risk individuals and support expectation management. Intraoperatively, careful positioning, neural preservation, limited unnecessary tissue disruption, protection of relevant sensory nerves, and selective anti-adhesion measures represent practical considerations rather than universally proven standards. Postoperative symptom screening can then guide stratified referral to voice, swallowing, or cervical rehabilitation pathways (9, 22, 24, 28, 36, 56, 60, 66). This model is consistent with contemporary surgery focused on patient-centered outcomes, but its effectiveness requires prospective validation.

**Figure 4:**
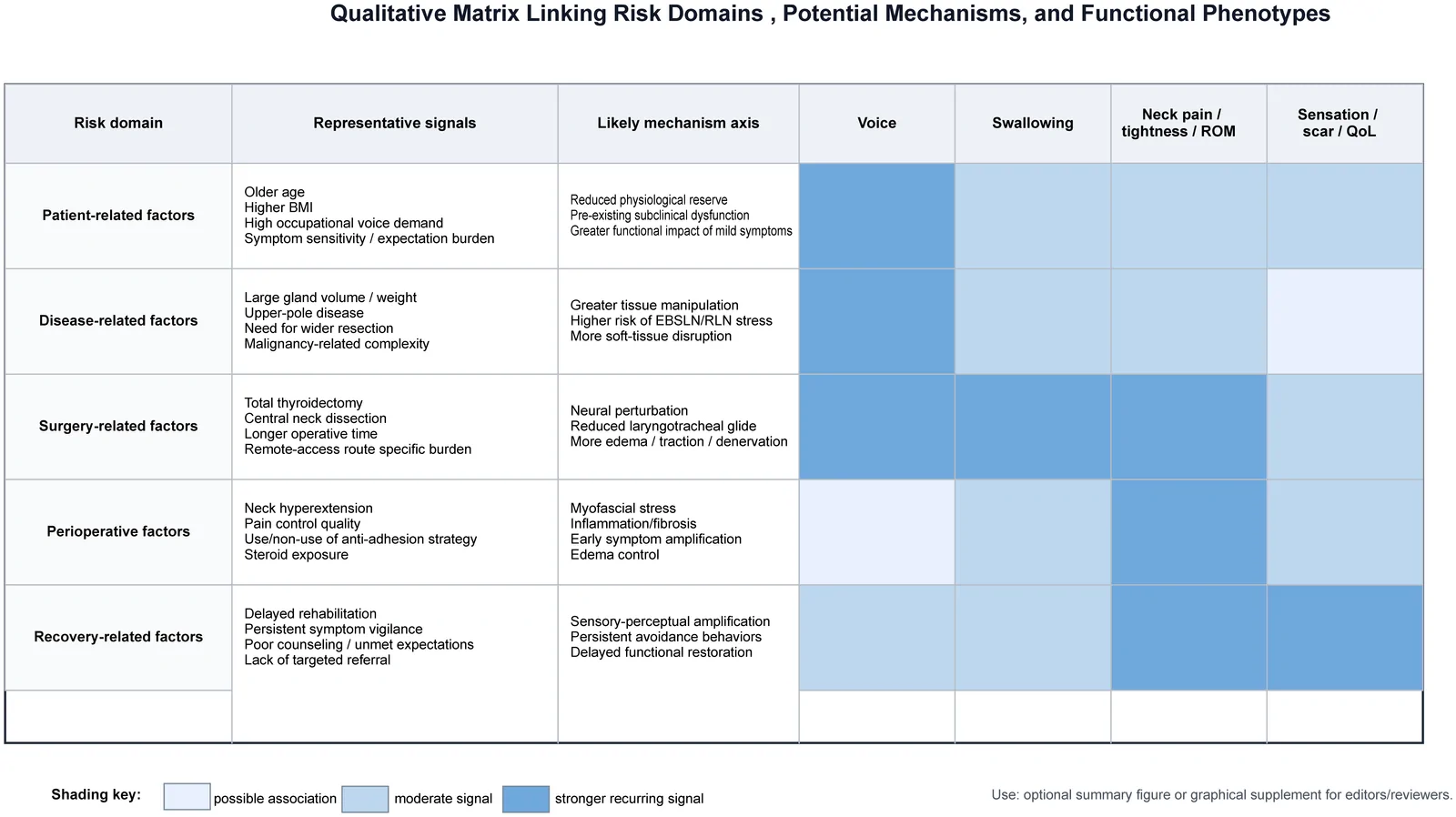
Qualitative matrix linking risk domains, potential mechanisms, and functional phenotypes. This qualitative matrix links major risk domains with potential mechanisms and functional phenotypes. The rows organize the literature into patient-related, disease-related, surgery-related, perioperative, and recovery-related domains. The middle column summarizes proposed mechanisms, and the shaded cells provide an illustrative impression of how often associations recur across the reviewed literature. The shading does not represent effect size, causality, or a formal certainty-of-evidence assessment.

Important limitations remain in both this review and the underlying evidence base. First, this is a narrative review based on a structured but non-systematic selection process; no prospective protocol, formal risk-of-bias tool, or quantitative pooling was used. The pragmatic journal-level filter applied during evidence prioritization cannot substitute for study-level quality assessment and may have introduced selection bias. Second, included studies are highly heterogeneous with respect to surgical approach, population, endpoint definition, assessment instrument, intervention protocol, and follow-up duration. Many intervention studies are small, single-center, or focused on short-term outcomes, limiting comparative inference and generalizability. Published correspondence on dysphagia in older adults further illustrates uncertainty regarding generalizability and risk attribution (58, 61). Third, there is no unified or externally validated definition of post-thyroidectomy anterior neck dysfunction; similar phenomena are described using different terms, and the framework proposed here requires prospective validation (3, 7, 26, 71). Fourth, most reported risk factors and mechanistic links are associative and do not establish causality. Finally, long-term studies that simultaneously address voice, swallowing, mobility, sensation, scar/adhesion, and quality of life remain scarce. **Figure 4** therefore presents a qualitative conceptual synthesis rather than a formal evidence grade or effect-size estimate.

## 10 Conclusions

The symptoms grouped in this review under post-thyroidectomy anterior neck dysfunction represent a real but under-recognized dimension of postoperative recovery. Rather than a formally established diagnosis, the term is proposed as a working framework for interrelated voice, swallowing, mobility, sensory, and scar/adhesion-related phenotypes that may arise from disruption of the anterior neck functional unit. Perioperative care may therefore benefit from extending beyond avoidance of major complications to include functional preservation, symptom-led assessment, and targeted rehabilitation. Prospective validation of the framework, standardized outcome measures, and adequately powered intervention studies are needed before uniform diagnostic or management recommendations can be established.
